# Reproducibility of Echocardiographic Measurements of Right Ventricular Function: Comparison Between TAPSE and TDI S′

**DOI:** 10.3390/jcdd13030104

**Published:** 2026-02-24

**Authors:** Gianluca Pagnoni, Dilia Giuggioli, Susan Darroudi, Marco de Pinto, Arianna Maini, Amelia Spinella, Gilda Sandri, Marcello Pinti, Daniela Aschieri, Alessio Baccarani, Anna Vittoria Mattioli, Francesco Fedele, Francesca Coppi

**Affiliations:** 1 Cardiology Unit of Emergency Department, Guglielmo da Saliceto Hospital, 29121 Piacenza, Italy; 2 Department of Medical and Surgical Sciences for Children and Adults, University of Modena and Reggio Emilia, Via del Pozzo 71, 41124 Modena, Italy; dilia.giuggioli@unimore.it (D.G.);; 3 Rheumatology Unit, Azienda Ospedaliero-Universitaria Policlinico di Modena, University of Modena and Reggio Emilia, 41121 Modena, Italy; marcodepinto1993@gmail.com (M.d.P.);; 4 Cardiology Division, Department of Biomedical Metabolic and Neural Sciences, University of Modena and Reggio Emilia, Via del Pozzo 71, 41124 Modena, Italy; 5 National Institute for Cardiovascular Research (INRC), Via Irnerio 48, 40126 Bologna, Italyannavittoria.mattioli@unibo.it (A.V.M.);; 6 Department of Life Sciences, University of Modena and Reggio Emilia, Via G. Campi 287, 41125 Modena, Italy; 7 Division of Plastic and Reconstructive Surgery, Department of Medical and Surgical Sciences, Modena Policlinico Hospital, University of Modena and Reggio Emilia, 41124 Modena, Italy; 8 Department of Quality of Life, University of Bologna-Alma Mater Studiorum, 40126 Bologna, Italy; 9 Emeritus of Cardiology, Sapienza University of Rome, 00185 Rome, Italy

**Keywords:** echocardiography, TAPSE, TDI, reproducibility

## Abstract

(1) Background: Assessment of right ventricular (RV) function has become increasingly important in echocardiography due to its prognostic significance in cardiac and systemic diseases. (2) Objectives: Tricuspid Annular Plane Systolic Excursion (TAPSE) and Tissue Doppler Imaging (TDI S′) are commonly used methods to assess RV function. The aim of this study was to compare the reproducibility of TAPSE and TDI S′ among experienced operators. (3) Methods: A study was conducted on 100 consecutive patients with systemic sclerosis undergoing echocardiography evaluation screening for pulmonary hypertension. Standardized echocardiography images were acquired using a Philips EPIQ system (Philips Healthcare, Amsterdam, The Netherlands), measuring TAPSE and TDI S′. Five operators independently analyzed the images, with each blinded to the results. Exclusion criteria included pathological TAPSE or TDI S′ values, as well as indirect echocardiography signs of pulmonary hypertension. (4) Results: The study’s results indicate that there was superior inter-operator agreement for TDI S′ (ICC = 0.891) compared to TAPSE (ICC = 0.47). The mean TAPSE measurements ranged from 23.49 mm to 23.99 mm, while TDI S′ values ranged from 12.16 cm/s to 12.35 cm/s, with TDI S′ showing narrower confidence intervals. (5) Conclusions: Echocardiographic assessment of right ventricular (RV) function is crucial in clinical practice. Tissue Doppler imaging-derived systolic velocity (TDI S′) demonstrated superior reproducibility compared to tricuspid annular plane systolic excursion (TAPSE) in evaluating RV function. This enhanced reproducibility supports the routine incorporation of TDI S′ as a highly reproducible parameter within a multiparametric assessment framework.

## 1. Introduction

The non-invasive assessment of right ventricular (RV) function is a fundamental component of standard echocardiographic examination due to the growing recognition of the prognostic relevance of RV dysfunction in various cardiac and systemic conditions, including pulmonary hypertension, myocardial infarction, valvular heart disease—particularly severe aortic stenosis in patients undergoing transcatheter aortic valve replacement (TAVR)—and heart failure [1,2,3,4,5]. The right ventricle, traditionally considered of secondary importance compared to the left, has now gained a central role in prognostic evaluation, as its dysfunction is associated with increased mortality and morbidity even in diseases not primarily affecting the pulmonary circulation [6,7,8].

Echocardiography represents the first-line tool for the functional evaluation of the right ventricle due to its availability, repeatability, and cost-effectiveness [2,9,10]. Among the most commonly used echocardiographic parameters for purpose are TAPSE [11] (Tricuspid Annular Plane Systolic Excursion), which quantifies the longitudinal systolic excursion of the tricuspid annulus, and the systolic velocity of the same annulus measured by Tissue Doppler Imaging (TDI S′) [12,13]. TAPSE is widely used in clinical practice for its simplicity and rapid acquisition; however, it is known to be highly dependent on the alignment of the ultrasound beam, image quality, and operator experience, all of which affect its reproducibility [11,13].

Tricuspid annular plane systolic excursion (TAPSE) is an M-mode measure of the longitudinal displacement of the lateral tricuspid annulus toward the apex during systole, typically obtained from an RV-focused apical four-chamber view with the M-mode cursor aligned with annular motion. It reflects predominantly longitudinal RV systolic shortening and is widely used due to its simplicity and feasibility. Tricuspid annular systolic velocity (S′) is derived from pulsed-wave tissue Doppler imaging (TDI) by placing the sample volume at the lateral tricuspid annulus to measure the peak systolic myocardial velocity; it similarly reflects longitudinal RV systolic function. Both parameters are influenced by loading conditions and require appropriate imaging alignment (particularly S′, which is angle-dependent), which can affect measurement variability [10].

Beyond the peak systolic annular velocity (S′), TDI allows systolic annular velocities (E′ and A′) and the derivation of time-interval-based indices such as the right ventricular myocardial performance index (RIMP/Tei index) and isovolumic acceleration (IVA), which have been proposed as complementary markers of global RV performance and contractility. However, these parameters may be less routinely acquired and can be more sensitive to technical factors and acquisition settings. For this reason, in the present reproducibility study, we focused on S′ as a simple, guideline-endorsed, and widely used TDI measure of RV systolic function [13,14].

However, few studies have systematically compared the agreement between these two measurements when assessed by different operators in a representative cohort of patients undergoing routine echocardiography, using standardized image acquisition protocols and involving operators with varying levels of experience.

The aim of the present study is to compare the reproducibility of TAPSE and TDI S′ measurements among five experienced operators, assessing agreement through advanced statistical analysis using the intraclass correlation coefficient. The study hypothesis is that TDI S′, due to its intrinsic characteristics, will demonstrate significantly higher inter-operator concordance compared to TAPSE.

## 2. Materials and Methods

A total of 100 consecutive patients with systemic sclerosis (scleroderma) undergoing routine echocardiographic evaluation at our cardiology center were enrolled as part of their first screening for pulmonary hypertension. This design was intentionally focused on a screening cohort with preserved RV systolic function and standardized image quality to isolate measurement-related variability. The homogeneity of the study population allowed for clinical standardization and minimized confounding variables in the assessment of right ventricular function. The study was approved by the local ethics committee of Area Vasta Emilia Nord (protocol no. 275/16) and was conducted in accordance with the Good Clinical Practice Guidelines and the World Medical Association Declaration of Helsinki, revised in 2000 (Edinburgh). All patients signed consent forms and were informed about the study.

Echocardiographic images were acquired using a Philips EPIQ system (Philips Healthcare, Amsterdam, The Netherlands), following a standardized protocol based on the guidelines of the American Society of Echocardiography. Specifically, our dataset was built from two standardized cardiac cycles acquired for each patient (M-mode for TAPSE and pulsed-wave TDI for S′). Measurements were performed offline by five independent readers, all blinded to each other’s results: one expert echocardiographer with >20 years of experience in transthoracic echocardiography, two final-year cardiology fellows who had already completed formal echocardiography training, and two sonographers with 2 years of echocardiography experience. All readers followed a standardized measurement protocol based on current echocardiography recommendations. This design allowed us to isolate the variability attributable to the measurement step alone, providing a reproducibility-focused assessment that, to the best of our knowledge, has not been previously addressed with this level of standardization.

The acquired images were subsequently analyzed by four additional operators: two certified sonographers and two cardiologists specialized in echocardiography. All operators independently performed measurements of both TAPSE and TDI S′, blinded to the values obtained by the others.

The exclusion of pathological TAPSE (<17 mm) and TDI S′ (<9.5 cm/s) values was a deliberate methodological choice to minimize biological variability related to overt RV dysfunction and to focus the analysis on measurement reproducibility.

All operators were previously trained in accordance with the standardized guidelines of the American Society of Echocardiography. Measurements were recorded in a centralized database, ensuring full anonymization and data traceability.

Statistical analysis was performed using SPSS version 25 to assess inter-operator agreement by calculating the intraclass correlation coefficient (ICC). Inter-operator agreement was assessed using the intraclass correlation coefficient (ICC). The prespecified primary reliability metric was the two-way random-effects model with absolute agreement for single measurements (ICC(2,1)), as both subjects and operators were considered random effects and absolute agreement was clinically relevant. In addition to ICC(2,1), other ICC formulations (ICC(1), ICC(3), and their corresponding average-measure versions ICC(k)) were computed for completeness and transparency. However, these additional models were not used for inferential conclusions and do not alter the primary interpretation based on ICC(2,1). Variance components were derived from a two-way random-effects ANOVA model, decomposing total variance into variance attributable to true between-subject differences, variance attributable to operators, and residual (unexplained) measurement error.

Mean values, standard deviations, and 95% confidence intervals (CI) were calculated for all measurements. Matrix scatter plots were generated to visually assess the reliability of TAPSE and TDI S′ measurements across operators. Agreement between methods was further evaluated using limits of agreement, calculated as the mean difference ± 1.96 times the standard deviation. Residual diagnostics and proportional bias analyses were performed for both TAPSE and TDI S′. Normality of residuals was assessed using quantile–quantile plots and the Shapiro–Wilk test. Homogeneity of variance was evaluated by plotting standardized residuals against the mean of both methods and tested using the Breusch–Pagan test. Proportional bias was examined by regressing residuals on the mean of the two methods, with a nonzero slope indicating linear bias. Residual analyses (including Shapiro–Wilk normality testing, Breusch–Pagan assessment of heteroscedasticity, and regression testing for proportional bias) were conducted as exploratory diagnostic procedures to evaluate agreement assumptions rather than as confirmatory hypothesis tests. Given the descriptive nature of these analyses, no formal adjustment for multiple comparisons was applied. Interpretation focused primarily on effect sizes, ICC magnitudes, and variance component estimates rather than isolated *p*-values. A two-sided *p*-value < 0.05 was considered statistically significant.

## 3. Results

A total of 100 participants were included in the study, comprising 84 (84%) females and 16 (16%) males. The mean age was 62 ± 12.16 years, with a mean BMI of 23.98 ± 3.99 kg/m^2^, body surface area of 1.68 ± 0.16 m^2^, and vitamin D level of 24.44 ± 11.43 nmol/L. Forty-four percent of participants were smokers, 12% were obese, and 10% had severe vitamin D deficiency. The prevalence of hypertension, diabetes mellitus, dyslipidemia, elevated uric acid (>7.5 mg/dL), and reduced estimated glomerular filtration rate (<45 mL/min/1.73 m^2^) was 40%, 6%, 75%, 15%, and 7%, respectively (Table 1).

**Figure 1:**
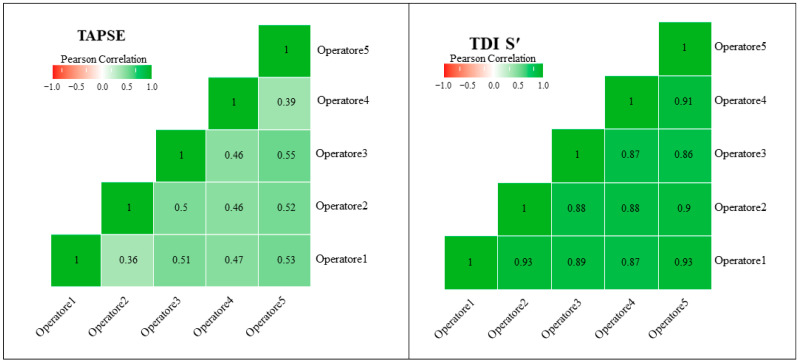
Heatmap of Inter-Rater Reliability Across Methods: TAPSE and TDI S.

Using the prespecified primary reliability metric (ICC(2,1)), the single-measure ICC for TAPSE was 0.473 (95% CI: 0.396–0.555), indicating poor reliability according to conventional thresholds (<0.5). Although average-measure reliability improved substantially (ICC(2,k) = 0.818), single-measure agreement remained limited, highlighting the potential impact of operator-dependent variability in routine clinical practice (Figure 1). When measurements were averaged across operators, reliability improved, with an ICC(2,k) of 0.818 (95% CI: 0.766–0.862), reflecting good group-level reliability (Table 2). Variance component analysis showed that between-subject variability accounted for 47.3% of total variance, while residual error constituted 52.7%, indicating substantial random measurement variability in TAPSE assessments (Table 3).

Inter-operator reliability for TDI S′ was significantly higher. Using the same ICC(2,1) model, single-measure inter-operator agreement for TDI S′ was 0.891 (95% CI: 0.863–0.915), indicating good-to-excellent reliability. The average-measures ICC further increased to 0.976 (95% CI: 0.969–0.982), indicating excellent reliability when measurements were averaged across operators (Table 2). Variance component analysis revealed that between-subject differences accounted for 89.1% of total variance, whereas between-operator variability was minimal (0.049%), with residual error contributing only 10.9% (Table 3) (Figure 1).

**Figure 2:**
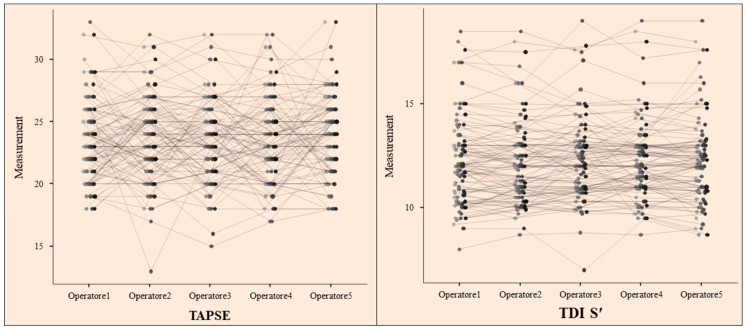
Individual Measurement Trajectories Across Raters: TAPSE vs. TDI S.

**Figure 3:**
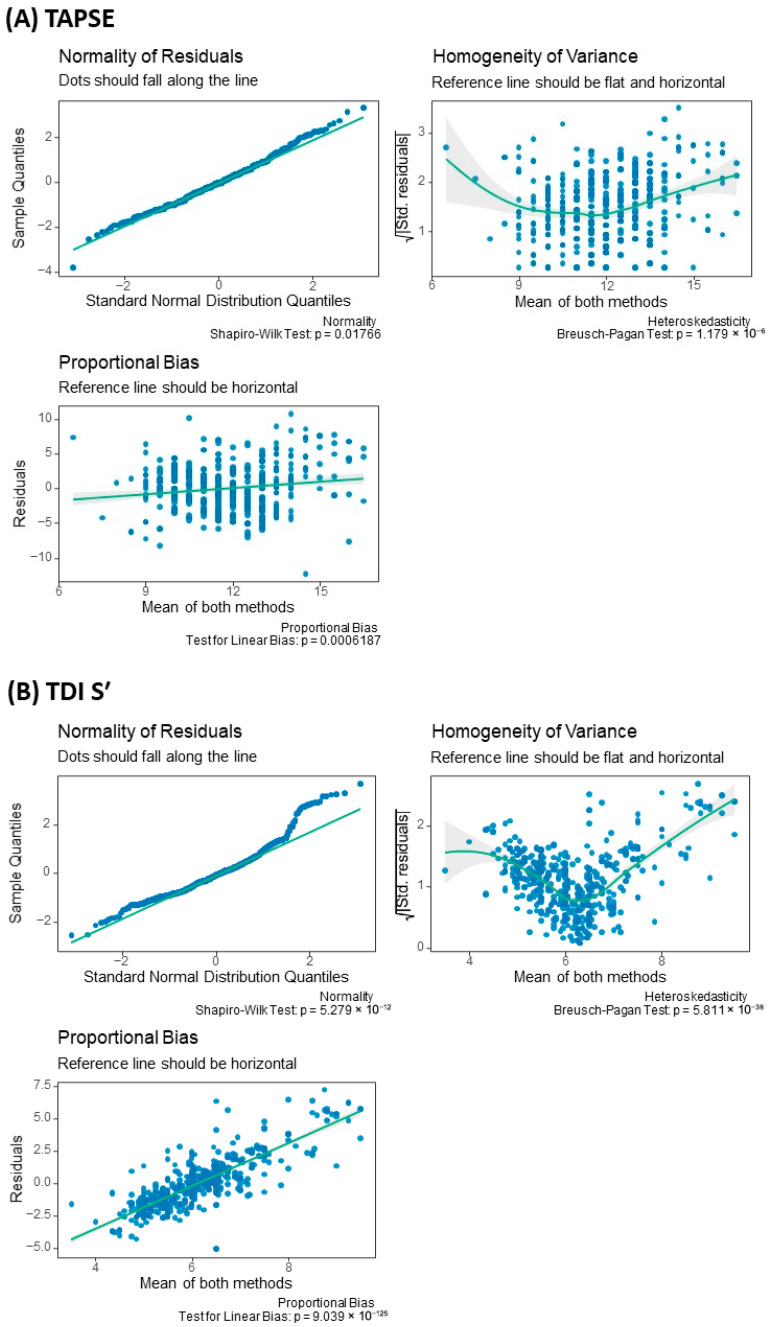
Residual Diagnostics and Proportional Bias Analysis for TAPSE and TDI S′ Measurements. (**A**) TAPSE and (**B**) TDI S′. Q–Q plots assess normality of residuals; blue points represent sample quantiles plotted against theoretical normal quantiles, and the solid line indicates the expected distribution under normality. Homogeneity of variance plots display the square root of absolute standardized residuals versus the mean of paired measurements; the solid line represents the fitted trend, and the grey shaded area indicates the 95% confidence interval. Proportional bias plots show residuals against the mean of paired measurements; the solid line represents the linear regression fit with corresponding 95% confidence band.

Assumption testing for Bland–Altman agreement analysis revealed distinct agreement characteristics between the two parameters. For TAPSE, residual analysis demonstrated significant heteroscedasticity (Breusch–Pagan *p* < 0.001) and proportional bias, with inter-operator differences increasing at higher measurement values (*p* < 0.001). These findings indicate that measurement variability is value-dependent rather than constant across the range of observations (Figure 2). Consequently, the conventional fixed limits of agreement derived from the standard Bland–Altman analysis should be interpreted with caution, as the assumption of homoscedastic error is not fully satisfied. Although Q–Q plots showed an approximately linear pattern, normality testing was statistically significant (Shapiro–Wilk *p* < 0.05). In contrast, TDI S′ demonstrated more favorable agreement properties, with stable variance across the measurement range. Although proportional bias testing may reach statistical significance, the slope was minimal (close to zero) and accounted for a negligible proportion of variability; the resulting change in inter-operator differences across the observed range was trivial and remained within the limits of agreement. Therefore, this finding was interpreted as not clinically meaningful (Figure 3).

Overall, comparison of the inter-observer agreement metrics demonstrated superior reproducibility and lower operator-dependent variability for TDI S′ compared with TAPSE. While reliability improved for both parameters when averaged across operators, TDI S′ maintained excellent agreement even at the single-measure level, supporting its greater clinical acceptability for routine assessment of right ventricular systolic function.

## 4. Discussion

TDI S′ demonstrated substantially higher inter-operator reliability than TAPSE in this standardized setting.

The present study demonstrates that tissue Doppler-derived tricuspid annular systolic velocity (TDI S′) provides substantially higher inter-operator reliability than tricuspid annular plane systolic excursion (TAPSE) for the assessment of right ventricular (RV) systolic function. These findings are highly consistent with the current American Society of Echocardiography (ASE) and European Association of Cardiovascular Imaging (EACVI) recommendations, which emphasize the importance of reproducible and operator-independent parameters in routine RV assessment, particularly in longitudinal follow-up and screening programs [2].

According to the ASE/EACVI guidelines, both TAPSE and TDI S′ are recommended indices of longitudinal RV systolic function; however, the guidelines explicitly acknowledge the limitations of TAPSE, including its angle dependence, reliance on precise M-mode cursor alignment, and susceptibility to translational motion of the heart [1]. These technical constraints inherently increase operator dependence and measurement variability, particularly in patients with suboptimal acoustic windows or altered thoracic anatomy [15]. Our findings directly support these guideline statements, as TAPSE demonstrated limited single-measure reliability, with improved agreement only when values were averaged across operators.

In contrast, ASE/EACVI guidelines describe TDI S′ as a robust and relatively load-independent Doppler-based parameter that reflects intrinsic myocardial systolic velocity. Because TDI S′ is less influenced by geometric assumptions and annular excursion artifacts, it is expected to exhibit superior reproducibility across operators. The excellent inter-operator reliability observed in our study for TDI S′ is consistent with guideline statements emphasizing the importance of reproducible parameters. However, current ASE/EACVI and ESC recommendations advocate a multiparametric approach to right ventricular assessment, in which TAPSE and TDI S′ are considered complementary rather than interchangeable indices [1,2].

The ESC guidelines on pulmonary hypertension and systemic connective tissue diseases further underscore the importance of reliable echocardiographic markers for the early detection of right ventricular dysfunction, particularly in systemic sclerosis and other chronic systemic conditions. In such populations, subtle and progressive RV impairment may precede overt hemodynamic deterioration [16,17]. Our results demonstrate that TDI S′ maintains stable agreement characteristics without proportional bias or heteroscedasticity, reinforcing its suitability for detecting early RV systolic dysfunction and for serial monitoring in at-risk patients, as advocated by ESC expert consensus documents.

Beyond reproducibility, TDI S′ has been shown in prior studies to correlate strongly with right ventricular ejection fraction derived from cardiac magnetic resonance imaging, which is regarded as the reference standard for RV functional assessment [18]. This strengthens the clinical relevance of TDI S′ as a surrogate marker, particularly in settings where advanced imaging modalities are unavailable, impractical, or contraindicated [19]. The current findings complement this evidence by demonstrating that TDI S′ not only reflects RV systolic performance but does so with minimal operator-dependent variability.

From a practical standpoint, ASE/EACVI guidelines emphasize the need for echocardiographic parameters that can be easily standardized across laboratories and operators. TDI S′ fulfills these criteria, as it is rapidly acquired, less affected by acoustic window quality, and less sensitive to probe orientation. These features enhance its feasibility in high-volume echocardiography laboratories, outpatient screening programs, and multicenter clinical studies [2,20]. Our results support this recommendation by demonstrating that the majority of variability in TDI S′ measurements is attributable to true inter-patient differences rather than operator-related error. However, from a clinical standpoint, it should be acknowledged that TAPSE remains one of the most widely used and best-validated surrogate measures for routine RV systolic assessment, given its feasibility and ease of standardization, while tricuspid annular TDI S′ represents a complementary, guideline-endorsed index that is also simple to acquire and generally reproducible when alignment is appropriate. Conversely, TDI-derived RIMP/MPI, although proposed as a global RV performance index, is more sensitive to methodological and physiologic factors (including the measurement of time intervals and conditions associated with elevated right-atrial pressure) and should be interpreted cautiously, rather than used as a stand-alone marker of global RV function [2].

The clinical implications of these findings are particularly relevant for longitudinal follow-up. In real-world practice, echocardiographic examinations are frequently performed by different operators over time. Parameters with high single-measure reliability, such as TDI S′, allow clinicians to distinguish true disease progression from measurement variability, thereby improving risk stratification, therapeutic decision-making, and the timing of further diagnostic evaluation [19,21].

Despite these strengths, several limitations should be acknowledged. The study population consisted exclusively of patients with systemic sclerosis undergoing outpatient screening, which may limit generalizability to other disease states or acute care settings. Additionally, excluding pathological TAPSE/TDI S′ values may limit the applicability of our reproducibility estimates to patients with advanced RV dysfunction, in whom both disease severity and poorer imaging conditions could further impact measurement variability. Furthermore, the study population mainly reflected a screening setting with preserved RV systolic function and standardized image quality. Therefore, our results may not be directly generalizable to patients with advanced RV dysfunction and/or suboptimal imaging conditions, in whom both reduced annular excursion and poorer acoustic windows may further affect measurement reproducibility. Cardiac magnetic resonance (CMR) is widely regarded as the reference standard for the quantification of RV volumes and systolic function. In the present study, CMR data were not systematically available; therefore, we could not assess the correlation of TAPSE or tricuspid TDI S′ with CMR-derived RV ejection fraction or RV volumetrics. Future studies should integrate CMR to validate whether the observed reproducibility differences translate into closer agreement with CMR-based RV functional assessment across broader clinical scenarios and imaging conditions. RA size/pressure surrogates and tricuspid regurgitation severity were not systematically available in the present dataset; future studies should assess whether RA remodeling and loading conditions influence the reproducibility of TAPSE and S′ in broader clinical populations, including those with significant LV disease. Finally, all measurements were obtained using a single echocardiographic platform (Philips Healthcare, Amsterdam, The Netherlands), and inter-vendor variability was not assessed. Nevertheless, these limitations do not detract from the internal validity of the reproducibility analysis. Future multicenter studies incorporating diverse patient populations, multiple ultrasound systems, and multimodality imaging comparisons are warranted to further validate and extend these findings.

## 5. Conclusions

In accordance with ASE/EACVI and ESC guideline principles, TDI S′ demonstrates superior inter-operator reliability and agreement characteristics compared with TAPSE. While a multiparametric approach to RV assessment remains recommended, our findings support the routine incorporation of TDI S′ as a highly reproducible parameter within a multiparametric framework for right ventricular assessment. However, it is reminded that these findings should be interpreted in the context of a screening cohort with preserved RV function and standardized imaging conditions.

## Figures and Tables

**Table 1:** Baseline Characteristics and Clinical Profile of the Study Population (*n* = 100).

Age, y		62 ± 12.16
Sex	Female	84 (84%)
	Male	16 (16%)
Smoking	No	56 (56%)
	Yes	44 (44%)
BMI, kg/m^2^		23.98 ± 3.99
Obesity	Normal	61 (61%)
	Overweight	27 (27%)
	Obese	12(12%)
BSA, m^2^		1.68 ± 0.16
Vitamin D, nmol/L		24.44 ± 11.43
Vitamin D deficiency	Severe	10 (10%)
	Moderate	25 (25%)
	Insufficient	41 (41%)
	Normal	24 (24%)
HTN	No	60 (60%)
	Yes	40 (40%)
DM	No	94 (94%)
	Yes	6 (6%)
Dyslipidemia	No	25 (25%)
	Yes	75 (75%)
UA > 7.5, mg/dL	No	85 (85%)
	Yes	15 (15%)
GFR < 45, mL/min/1.73 m^2^	No	93 (93%)
	Yes	7 (7%)

Data are presented as mean ± SD or number (%). Abbreviations: BMI, body mass index; BSA, body surface area; HTN, hypertension; DM, diabetes mellitus; UA, uric acid; GFR, glomerular filtration rate. Units: Age (years); BMI (kg/m^2^); BSA (m^2^); vitamin D (nmol/L).

**Table 2:** Intraclass Correlation Coefficients for Inter-Operator Reliability of TAPSE and TDI S′ (Primary Model: ICC(2,1)).

Model	Measures	Type	ICC	Lower C.I.	Upper C.I.
**TAPSE**					
One-way random	Agreement	ICC1	0.473	0.396	0.555
Two-way random	Agreement	ICC2	0.473	0.396	0.555
Two-way fixed	Consistency	ICC3	0.473	0.396	0.555
One-way random	Avg. Agreement	ICC1k	0.818	0.766	0.862
Two-way random	Avg. Agreement	ICC2k	0.818	0.766	0.862
Two-way fixed	Avg. Consistency	ICC3k	0.818	0.766	0.862
**TDI S′**					
One-way random	Agreement	ICC1	0.891	0.863	0.915
Two-way random	Agreement	ICC2	0.891	0.863	0.915
Two-way fixed	Consistency	ICC3	0.891	0.864	0.916
One-way random	Avg. Agreement	ICC1k	0.976	0.969	0.982
Two-way random	Avg. Agreement	ICC2k	0.976	0.969	0.982
Two-way fixed	Avg. Consistency	ICC3k	0.976	0.969	0.982

Intraclass Correlation coefficients were calculated using one-way random, two-way random (absolute agreement), and two-way mixed (consistency) models. The prespecified primary reliability metric was the two-way random-effects absolute agreement model for single measurements (ICC(2,1)). Additional ICC forms are presented for transparency but do not change the interpretation of inter-operator agreement.

**Table 3:** Variance Components for TAPSE and TDI S′.

TAPSE			TDI S′		
Component	Variance	Percent	Component	Variance	Percent
Subject variance	5.09	0.473	Subject variance	3.56262	0.891
Operator variance	7.05 × 10^−10^	6.56 × 10^−11^	Operator variance	0.00196	4.91 × 10^−4^
Residual error	5.66	0.527	Residual error	0.43476	0.109
Total	10.75	1.000	Total	3.99935	1.000

Variance components were estimated using a two-way random-effects model to assess inter-operator measurement variability. “Subject variance” represents variance attributable to true subject-level differences. “Operator variance” corresponds to operator-related variance, and “Residual error” reflects unexplained variance (random error). A higher proportion of variance attributed to “Subject variance” indicates greater measurement consistency across operators.

## Data Availability

The data presented in this study are available on request from the corresponding authors.
